# CGTV-Tm: A High-Accuracy Gridded Atmospheric Weighted Mean Temperature Model Coupling Surface Temperature and Water Vapor Pressure over China

**DOI:** 10.3390/s26134218

**Published:** 2026-07-03

**Authors:** Yaoshuang Zhang, Jian Mao

**Affiliations:** Faculty of Geography, Tianjin Normal University, Tianjin 300387, China; zhangys@stu.tjnu.edu.cn

**Keywords:** atmospheric weighted mean temperature, GNSS meteorology, vertical correction, daily weather forecast data, precipitable water vapor

## Abstract

The atmospheric weighted mean temperature ($T_{m}$) is critical for converting a zenith wet delay (ZWD) to precipitable water vapor (PWV). However, the existing $T_{m}$ models still have certain limitations: Those driven by surface-measured parameters achieve high accuracy but depend heavily on in situ instruments, incurring high costs and lacking forecasting capability. Empirical models avoid measured data but fail to capture short-term $T_{m}$ variations, leading to lower accuracy. Daily weather forecast data—which are low-cost, readily available, and reflective of short-term changes—offer a promising alternative. This study develops a gridded $T_{m}$ model named CGTV-Tm, which couples temperature and water vapor pressure, using ERA5 reanalysis data over China (2019–2023). The model can be driven by daily weather forecast data. A dual vertical correction method is also proposed to improve performance. Validation against 2024 ERA5 and radiosonde data shows that CGTV-Tm achieves RMSEs of 2.38 K (vs. ERA5) and 2.64 K (vs. radiosonde), significantly outperforming the Bevis (3.61 K, 3.67 K), PTm (3.19 K, 2.94 K), and CGT-Tm (2.71 K, 3.08 K) models. When driven by daily weather forecast data, CGTV-Tm achieves an RMSE of 2.90 K, improving accuracy by 29.6% and 21.2% over the state-of-the-art empirical models GPT3 and HGPT2, respectively. These results demonstrate that CGTV-Tm not only surpasses traditional linear $T_{m}$ models that rely solely on surface temperature but also, by using weather forecast data, it removes dependence on in situ instruments, offering a superior low-cost solution for real-time GNSS (Global Navigation Satellite System) PWV retrieval.

## 1. Introduction

The accurate acquisition of precipitable water vapor (PWV) data is of great significance for weather forecasting, climate change research, and extreme precipitation disaster monitoring. Retrieving PWV via Global Navigation Satellite System (GNSS) technology has become the primary approach to acquiring PWV data with a high spatiotemporal resolution. The prerequisite condition of this technique is the zenith tropospheric delay (ZTD) generated when GNSS signals pass through a neutral atmosphere. A ZTD can be decomposed into a zenith hydrostatic delay (ZHD) and zenith wet delay (ZWD). Askne and Nordius derived an approximate relationship between a ZWD and PWV, laying the theoretical foundation for GNSS PWV retrieval [1]. Bevis further proposed using a water vapor conversion factor (∏) to retrieve PWV from a ZWD [2]. Among all the parameters in the water vapor conversion factor (∏), the atmospheric weighted mean temperature ($T_{m}$) is the only meteorological variable that significantly varies with location and time, while the other terms are physical constants.

Obtaining the true $T_{m}$ requires water vapor pressure and temperature profiles. Currently, such data are primarily obtained from radiosondes and reanalysis datasets. However, while radiosondes provide high observational accuracy, they are typically released only twice daily, and their locations often do not coincide with GNSS stations. On the other hand, reanalysis data suffer from time lags and thus cannot fulfill the requirements of real-time GNSS PWV retrieval. Therefore, the use of models to calculate $T_{m}$ has become the primary approach.

In 1992, Bevis et al. utilized radiosonde data from the United States and first discovered a strong linear relationship between $T_{m}$ and surface temperature ($T_{s}$). Based on this finding, they established the well-known Bevis linear regression formula, which laid the foundation for the method of obtaining $T_{m}$ in early GNSS PWV retrieval [2]. However, since this model was developed using meteorological data from the mid-latitude regions of the United States, its direct application to other regions results in significant systematic biases. The latitudinal dependence of the $T_{m}$-$T_{s}$ correlation has long been recognized as a key factor in $T_{m}$ modeling [3], and has subsequently spurred the development of numerous regional models. These include $T_{m}$ models for China [4,5], for Europe [6], for Hong Kong [7], for Canada and Alaska [8], for India [9], for Algerian [10], for Brazilian [11], for the Arabian Peninsula [12], and for polar regions [13]. With the widespread application of reanalysis data, Yang et al. utilized ECMWF data from 2011 to 2015 to construct a global $T_{m}$-$T_{s}$ relationship model with a spatial resolution of 2.5° × 2°. This model preserves the regional characteristics of $T_{m}$ while enabling global applications [14]. However, most of these models are linear models that only consider the single factor of surface temperature. According to the results of Yao et al. [15], the accuracy of a nonlinear global unified model that incorporates surface water vapor pressure is superior to that of a linear global unified model. Unfortunately, after gridding, the accuracy of the nonlinear model becomes inferior to that of the linear model.

Leveraging ground-measured meteorological parameters, the above models can generally achieve satisfactory accuracy in $T_{m}$ estimation. However, numerous GNSS stations worldwide are not equipped with in situ meteorological instruments, making it difficult to meet the requirements for real-time GNSS PWV retrieval. To overcome this limitation, empirical $T_{m}$ models that require only spatial and temporal information—without the need for measured meteorological parameters—have emerged, representing another important direction in $T_{m}$ modeling research. The UNB3m model is one of the representative early achievements [16]. Although this model is primarily used for tropospheric delay correction, its intermediate parameter $T_{m}$ can be calculated from the meteorological parameters predicted using the station’s latitude, elevation, and day of year, which laid the foundation for the parameterization approach based on latitude and seasonal cycles. From 2012 to 2014, Yao et al. proposed the GTm series of models. Both GTm I and GTm II were constructed jointly using the GPT model and radiosonde data, considering only annual periodic variations with a spatial resolution of 10° × 20°; GTm II overcame the issue of the insufficient prediction accuracy of GTm I over oceanic regions [17,18]. GTm III further introduced semi-annual and diurnal periodic characteristics and estimated the initial phases for each period, achieving better performance on a global scale than previous models [19]. Subsequently, Böhm et al. developed the GPT2w model based on monthly mean pressure-level data from ERA-Interim, providing the annual mean value of $T_{m}$ and annual/semi-annual amplitudes at a horizontal resolution of 1° [20]. In 2018, Landskron and Böhm further released the GPT3 model, which has become one of the most widely used global empirical models [21]. Mateus et al. proposed the HGPT2 model based on 20 years of ERA5 reanalysis data, with a spatial resolution of 0.25° × 0.25° and a temporal resolution of 1 h [22]. As spatiotemporal resolutions continue to improve, the lack of vertical correction capabilities in empirical models has become increasingly prominent. To address this shortcoming, Yang et al. utilized 10 years of ECMWF monthly pressure-level data to explore the $T_{m}$ lapse rate [23]. Later, models such as the GGTm-H [24], IGTmS [25], and NGGTm-H were successively proposed [26], effectively enriching and expanding the $T_{m}$ model system. However, empirical models are essentially statistical models based on climatological averages; they reflect the long-term variation patterns of $T_{m}$ but struggle to capture its short-term characteristics.

In summary, models based on surface-measured meteorological parameters achieve high prediction accuracy, but their performance heavily depends on in situ meteorological instruments, which not only increases construction and maintenance costs but also limits real-time PWV retrieval at GNSS stations without such sensors. In contrast, empirical models overcome this problem; however, due to their difficulty in capturing short-term variations, their accuracy is relatively low. Daily weather forecast data are characterized by low costs, easy accessibility, and the ability to reflect short-term variations in meteorological parameters; however, their application in GNSS PWV retrieval has not yet been reported. Therefore, this study aims to develop a $T_{m}$ model that can break free from dependence on in situ meteorological instruments and achieve a high-accuracy forecasting capability, using daily weather forecast data as the input. To this end, focusing on China as the study area, we first analyzed the impact of different combinations of surface meteorological parameters on $T_{m}$ prediction accuracy, and subsequently developed a gridded $T_{m}$ model coupling surface temperature and water vapor pressure over China, referred to as CGTV-Tm. Meanwhile, to enhance the performance of CGTV-Tm, a dual vertical correction method was proposed. For comparison with traditional modeling approaches, a gridded $T_{m}$ model depending solely on surface temperature over China, named CGT-Tm, was also developed. Finally, using $T_{m}$ derived from radiosonde and using ERA5 reanalysis data as a reference, the effectiveness of the CGTV-Tm model was systematically evaluated under two scenarios: one driven by surface-measured meteorological parameters and one driven by daily weather forecast data.

## 2. Data and Methods

### 2.1. Data Sources

#### 2.1.1. ERA5 Reanalysis Data

ERA5 is the fifth-generation global atmospheric reanalysis product released by the European Centre for Medium-Range Weather Forecasts (ECMWF). It employs an advanced four-dimensional variational data assimilation (4D-Var) technology technique and a variety of observational data to provide a consistent, high-quality grid-based reconstruction of historical meteorological conditions. Compared to previous generations (such as ERA-Interim), ERA5 offers significant improvements in spatial and temporal resolution, the richness of variables, and assimilation performance. The product covers the period from 1940 to the present, providing gridded atmospheric state variables across 37 pressure levels ranging from 1000 hPa to 1 hPa. With a spatial resolution of 0.25° × 0.25° and a temporal resolution of 1 h, it is capable of finely capturing the spatiotemporal evolution of atmospheric dynamic and thermal processes. This study utilized ERA5 pressure-level and surface data for the Chinese region from 2019 to 2023 for model construction, and employed 2024 data for model validation.

#### 2.1.2. Radiosonde Data

**Figure 1:**
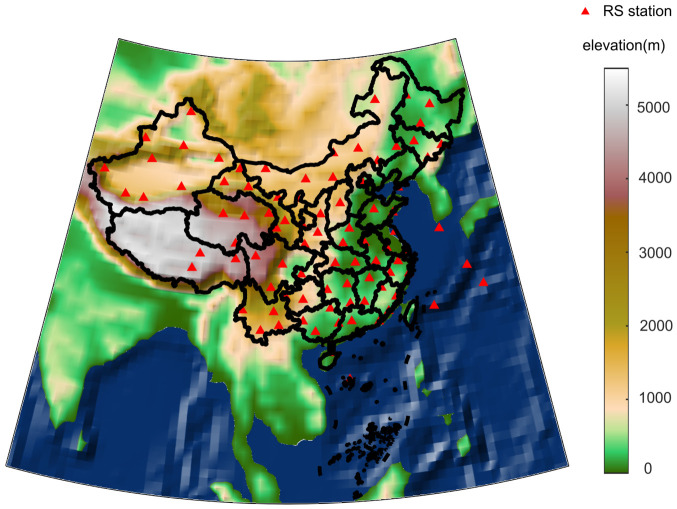
Geographic distribution and terrain elevation of RS (Radiosonde) stations in China.

Radiosonde data are important observational records that utilize sounding balloons equipped with high-precision meteorological sensors to obtain information on the vertical structure of the atmosphere during their ascent. Sounding balloons are typically released at 00:00 and 12:00 (UTC) each day, continuously measuring the vertical profiles of pressure, temperature, relative humidity, and geopotential height. Such data feature high vertical resolution and measurement accuracy, and are widely regarded as the true representation of atmospheric vertical structure. They are commonly used for the validation and error correction of reanalysis products and numerical weather prediction models. In this study, radiosonde data from 89 stations over China in 2024, provided by the University of Wyoming’s sounding website, are adopted as reference for model validation. The geographical distribution of the radiosonde stations is illustrated in Figure 1. It should be noted that all 89 radiosonde stations are used for the validation of models driven by measured meteorological parameters, while the forecast-data-driven validation involves only 83 stations that coincide with the locations of the 83 forecast cities.

#### 2.1.3. Daily Weather Forecast Data

**Figure 2:**
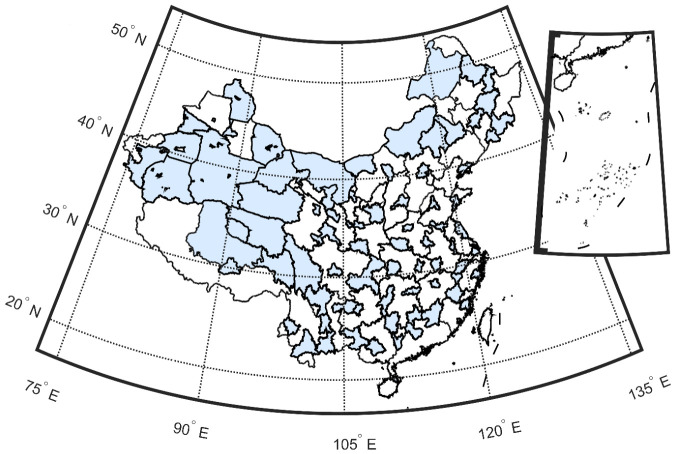
Geographical coverage of daily weather forecast data for 83 prefecture-level cities (shaded in blue) in China.

Daily weather forecast data, characterized by low costs, high timeliness, easy accessibility, and extensive spatial coverage, provide a highly attractive data source for GNSS water vapor retrieval. Applying these data to $T_{m}$ calculations can effectively address the multiple limitations of traditional methods, including the strong dependence on specialized equipment, insufficient real-time capability, and the inability of empirical models to capture short-term $T_{m}$ variations. This opens up new possibilities for low-cost real-time PWV monitoring, extreme weather early warnings, and climate change research. In this study, the CGTV-Tm model is driven by daily weather forecast products released by the China Meteorological Administration (CMA). These forecast products are generated by the self-developed CMA-MESO mesoscale system, which assimilates multi-source observations such as Fengyun satellite data, radar, surface measurements, and radiosonde data. Through data assimilation and model integration, initial and forecast fields are produced and subsequently refined by manual correction techniques to generate the final forecast products [27,28,29,30]. We regularly downloaded daily weather forecast data for 2024 from 83 prefecture-level cities in China from the official CMA website (http://weather.cma.cn/). The geographic locations of these cities are shown in Figure 2. The CMA-MESO mesoscale model operates eight times daily with a 3-hourly update cycle (initiated at 00:00, 03:00, 06:00, 09:00, 12:00, 15:00, 18:00, and 21:00 UTC). In practice, we employed only the forecast cycle initialized at 00:00 UTC, and from this single cycle we extracted forecasts at 3-hourly intervals (i.e., at 03, 06, 09, …, 24 h after initialization), corresponding to a 24 h lead time. For comparison with radiosonde data, forecast values were assigned to the radiosonde observation times (00:00 and 12:00 UTC) using the nearest available forecast hour.

### 2.2. Methods

When electromagnetic signals emitted by GNSS satellites propagate through the neutral atmosphere, they experience delays due to the effects of temperature, atmospheric pressure, and water vapor; this delay is known as tropospheric delay. Typically, the value of a tropospheric delay is calculated by multiplying the zenith tropospheric delay (ZTD) by a mapping function. A ZTD can be further decomposed into two components: the zenith hydrostatic delay (ZHD), caused by atmospheric gases in a hydrostatic equilibrium state, and the zenith wet delay (ZWD), caused by atmospheric gases in a non-hydrostatic equilibrium state. The relevant calculations are:

(1) ZTD = ZWD + ZHD,

In GNSS meteorology, a ZTD is often estimated as a parameter with an accuracy of up to 4 mm [31], while a ZHD is calculated using the Saastamoinen formula [32].

(2) $$ZHD=\frac{2.2768P_{S}}{1-0.00266COS2\lambda -0.00000028H},$$

where $P_{s}$ is the station pressure, λ is the station latitude, and $H^{\prime }$ is the station elevation (meters). The ZHD values calculated using this formula can achieve millimeter-level accuracy [2]. Based on this, a ZWD can be separated from a ZTD using Equations (1) and (2). To further retrieve PWV from a ZWD, Bevis et al. proposed a conversion model based on the approximate relationship between a ZWD and PWV derived by Askne and Nordius [1,2]:

(3) $$PWV=\frac{10^{6}}{\rho_{w}R_{v}(\frac{k_{3}}{T_{m}}+k_{2}^{\prime })}\cdot ZWD,$$

where $\rho_{w}$ denotes the density of liquid water, with a value of 1000 kg/m^3^; $R_{v}$ denotes the specific gas constant of water vapor, with a value of 461.5 J/kg/K; $k_{2}^{\prime }$ and $k_{3}$ are atmospheric refractive index constants; and $T_{m}$ denotes the atmospheric weighted mean temperature. As can be seen from Equation (3), $T_{m}$ is the sole variable determining the accuracy of the water vapor retrieval. It is defined by Davis as [33]:

(4) $$T_{m}=\frac{\int_{H}^{\infty }e/Tdz}{\int_{H}^{\infty }e/T^{2}dz},$$

where H is the surface height, e is the water vapor pressure, and T is the temperature. The water vapor pressure can be calculated from the relative humidity (RH) and the saturated water vapor pressure $e_{sat}$ [34]:

(5) $$e=RH*e_{sat}/100,$$

(6) $$e_{sat}=e^{\left(1.2378847*10^{-5}T^{2}+-1.9121316*10^{-2}T+33.93711047+\frac{-6.3431645*10^{3}}{T}\right)},$$

when the measured data are the dew-point temperature $T_{dew}$, it can be calculated directly using the Magnus formula [35]:

(7) $$e=6.112*exp\left(\frac{17.62*T_{dew}}{T_{dew}+243.12}\right),$$

Since meteorological parameters in both radiosonde and reanalysis data are stored in discrete layers, $T_{m}$ can be calculated using discrete integration [36]:

(8) $$T_{m}=\frac{\sum_{i=1}^{n}\frac{e_{i}}{T_{i}}*\Delta H_{i}}{\sum_{i=1}^{n}\frac{e_{i}}{{T^{2}}_{i}}*\Delta H_{i}},$$

where n represents the total number of meteorological layers, and $e_{i}$, $T_{i}$, and ${\Delta H}_{i}$ denote the mean vapor pressure, mean temperature, and atmospheric thickness of the i-th layer, respectively.

Given that the profiles of temperature and water vapor pressure near GNSS stations are difficult to obtain, many researchers have turned to using more easily accessible surface meteorological parameters to estimate $T_{m}$. Bevis et al. based on the good linear correlation between surface temperature ($T_{s}$) and $T_{m}$, established the first global linear $T_{m}$-$T_{s}$ model, known as the Bevis model [2]:

(9) $$T_{m}^{Bevis}=70.2+0.72T_{s},$$

On this basis, Yao et al. (2013) further introduced water vapor pressure and the proposed the PTm model [15]:

(10) $$T_{m}^{PTm}=81.9+0.5344T_{s}+31.81e_{s}^{0.1131},$$

These two models are typical representatives of surface-measured meteorological parameter models. As for empirical models, the most widely used ones currently are the GPT3 and HGPT2 models. The GPT3 model stores $T_{m}$ in a 1° × 1° global grid in the form of annual mean ($a_{0}$), annual amplitudes ($a_{1}$, $b_{1}$), and semi-annual amplitudes $(a_{2}$, $b_{2}$). Its specific expression is as follows:

(11) $$T_{m}^{GPT3}=a_{0}+a_{1}\cos\left(2\pi \frac{DOY}{365.25}\right)+b_{1}\sin\left(2\pi \frac{DOY}{365.25}\right)+a_{2}\cos\left(4\pi \frac{DOY}{365.25}\right)+b_{2}sin(4\pi \frac{DOY}{365.25}),$$

where DOY denotes days of year. The HGPT2 model is based on the linear relationship between $T_{s}$ and $T_{m}$. It stores the parameters α and β of the linear $T_{m}$-$T_{s}$ function, as well as the annual mean, annual period, semi-annual period, and seasonal variation period of hourly $T_{s}$, in a global $0.25^{\circ }\times 0.25^{\circ }$ grid. The specific expressions are as follows:

(12) $$T_{m}^{HGPT2}=\alpha +\beta T_{s},$$

(13) $$T_{s}^{h}\left(t\right)=a^{h}+b^{h}\cdot \left(t-t_{0}\right)+a_{1}^{h}\cdot \cos\left(\frac{2\pi \cdot \left(t-t_{0}\right)}{365.25}+f_{1}^{h}\right)+a_{2}^{h}\cdot cos\left(\frac{2\pi \cdot \left(t-t_{0}\right)}{182.63}+f_{2}^{h}\right)+a_{3}^{h}\cdot \cos\left(\frac{2\pi \cdot \left(t-t_{0}\right)}{91.31}+f_{3}^{h}\right),$$

where t is the Modified Julian Day (MJD), $t_{0}$ is the date of the first observation, and h is the h-th hour of day t (UTC); a and b are the regression coefficients; $a_{1}$, $a_{2}$, and $a_{3}$ are the annual, semi-annual, and quarterly amplitudes, respectively; and $f_{1}$, $f_{2}$, and $f_{3}$ are the initial phases for the annual, semi-annual, and quarterly components, respectively.

## 3. Development of the CGTV-Tm Model

### 3.1. Theoretical Basis of Model Development

The introduction of daily weather forecast data represents a solution to address heavy reliance on in situ meteorological instruments for surface-measured meteorological parameter $T_{m}$ models, as well as the inability of empirical models to capture short-term $T_{m}$ variations. However, such data provide short-term predictions of the surface atmospheric state over a regional area, which inevitably differ from the actual atmospheric state at a specific GNSS station. Therefore, it is essential to first establish a model that can accurately reflect the relationship between local surface meteorological parameters and $T_{m}$.

In meteorology, atmospheric stratification refers to the vertical layered state of the atmosphere, which is determined by the distributions of temperature and humidity, and particularly by the variation of temperature with height (i.e., the temperature lapse rate) [37]. According to Equation (4), $T_{m}$ is closely related to temperature, water vapor pressure, and their vertical lapse rates. Therefore, it can be inferred that the variability of atmospheric stratification directly affects the prediction accuracy of $T_{m}$ models that rely on surface meteorological parameters. The temporal alternation of different stratification states is primarily driven by the diurnal variation of surface net radiation. Although water vapor content does not determine whether such alternation occurs, it significantly influences the intensity, timing, and vertical structure of stratification transitions by modulating radiative cooling, latent heat release, and cloud formation [38]. In dry climate regions, stratification alternation tends to be more intense and structurally distinct, whereas in humid regions, the transitions are relatively smooth and more easily disturbed by clouds and latent heating processes [39]. Thus, both temperature and water vapor jointly regulate the variation characteristics of atmospheric stratification. Consequently, in addition to surface temperature, the effect of surface water vapor pressure on $T_{m}$ must also be considered.

Yao et al. (2013) demonstrated that the $T_{m}$ model incorporating water vapor pressure (PTm) outperforms the model relying solely on surface temperature (GTm) in terms of accuracy [15]. However, when a residual correction with a spatial resolution of $2^{\circ }\times 2.5^{\circ }$ was introduced to PTm (denoted as PTm-I), its accuracy became lower than that of the similarly corrected GTm model (denoted as GTm-I). Given that the spatiotemporal variability of water vapor is much greater than that of temperature, we suggest that the $2^{\circ }\times 2.5^{\circ }$ spatial resolution is insufficient to capture the spatial variation characteristics of water vapor, which may be the primary reason why PTm-I underperforms GTm-I after residual correction. In recent years, the spatial resolution of ERA5 reanalysis data has continued to improve, creating favorable conditions for enhancing the accuracy of $T_{m}$ models based on water vapor pressure. Additionally, daily weather forecast data generally lack height information, while height is closely related to $T_{m}$. How to effectively address height correction during the modeling process presents another challenge.

### 3.2. Core Model Development

**Figure 3:**
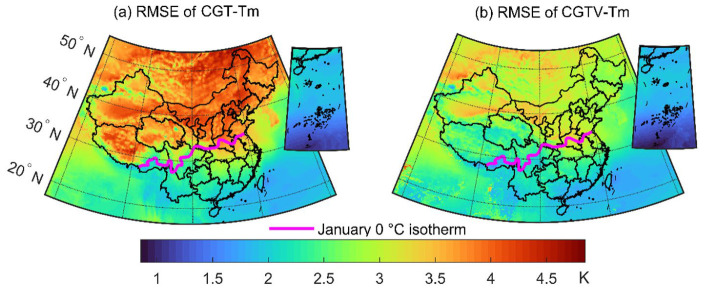
Spatial distribution of the root mean square errors (RMSEs) for the CGTV-Tm and CGT-Tm models.

Based on the modeling strategy outlined above and referencing the PTm model, we express the relationship between $T_{m}$, $T_{s}$, and $e_{s}$ in a nonlinear form. That is:

(14) $$T_{m}=A+B\times T_{s}+C{e_{s}}^{D},$$

where A, B, C, and D are model coefficients. For comparison with traditional linear models that rely solely on surface temperature and as a complement to the nonlinear model, we also develop a relationship model between $T_{m}$ and surface temperature:

(15) $$T_{m}=\delta +\gamma T_{s},$$

where δ and γ are model coefficients. Subsequently, this study first extracted temperature and water vapor pressure profile data from a total of 205 × 253 grid points within the Chinese region (73° E–136° E, 3° N–54° N) using ERA5 pressure-level and surface data with a horizontal resolution of 0.25° × 0.25° for the period 2019–2023. Then, the surface $T_{m}$ at each grid point was calculated according to Equation (8), and the corresponding $T_{s}$ and $e_{s}$ data were extracted. Finally, based on these data, the least squares method was applied to fit Equations (14) and (15), respectively, thereby establishing a regional $T_{m}$ model over China that couples surface temperature and water vapor pressure (referred to as CGTV-Tm) and a $T_{m}$ model relying solely on surface temperature (referred to as CGT-Tm). Figure 3 shows the spatial distribution of the root mean square error (RMSE) of the fitted results for the CGTV-Tm and CGT-Tm models. The red line in the figure represents the 0 °C isotherm in January, which serves as a climatic dividing line in China. South of this line lies the subtropical monsoon climate, characterized by high temperatures and high humidity in summer, and mild, relatively humid winters with moderate temperature variations. North of the line are the warm-temperate and mid-temperate monsoon climates, which, compared to the subtropical monsoon climate, exhibit significantly lower water vapor content, more drastic temperature variations, and a sharper contrast between dry and wet conditions.

As shown in Figure 3, the RMSE of both the CGTV-Tm and CGT-Tm models exhibits a gradually increasing trend from low to high latitudes, with high-value areas mostly located north of the January 0 °C isotherm, indicating that the latitudinal zonality of the atmospheric thermal structure may be the dominant factor controlling the errors. This is because low-latitude regions, influenced by the Intertropical Convergence Zone (ITCZ), have abundant atmospheric water vapor and a relatively stable thermal structure, with a gentle vertical temperature lapse rate, leading to smaller temporal variability in $T_{m}$ and thus enabling high-precision prediction by the models. In contrast, mid- to high-latitude regions, affected by westerly wave disturbances and land–sea thermal contrasts, exhibit large diurnal temperature ranges, coupled with lower water vapor content and its uneven spatiotemporal distribution, resulting in a more complex vertical atmospheric structure, often accompanied by pronounced stratification changes such as multiple inversion layers. This complexity leads to strong nonlinearity and seasonal fluctuations in $T_{m}$, significantly increasing the difficulty of model prediction. This phenomenon is more pronounced for the CGT-Tm model, where the fitting error increases sharply with latitude, reaching 3.5–4.5 K north of 40° N, reflecting that the simple linear relationship fails to capture the complex structures (e.g., frequent inversions and dry/wet multilayers) in northern regions. In contrast, after incorporating surface water vapor pressure, the CGTV-Tm model significantly reduces the RMSE in northern China to 2.5–3.0 K, while maintaining low values of 1.5–2.0 K in the low latitudes. This improvement occurs because surface water vapor pressure acts as an effective proxy for atmospheric structural complexity, enabling the model to distinguish between the “low temperature, high humidity” inversion-stabilized state and the “low temperature, low humidity” dry adiabatic state, thereby providing critical vertical stratification information and substantially enhancing the fitting accuracy of $T_{m}$ under complex atmospheric conditions at mid-to-high latitudes.

### 3.3. Vertical Correction Method for the Model

Previous studies have shown that height significantly affects the estimation accuracy of $T_{m}$ models [23,24,25,26]. The CGTV-Tm and CGTV-Tm models are both developed based on the surface height of ERA5 data, while the heights of GNSS sites often differ from these reference heights. Directly using meteorological data observed at the site as the model input would introduce errors, especially for daily weather forecast data that lack height information. Therefore, it is necessary to incorporate vertical correction into the models. Given that the CGTV-Tm and CGTV-Tm models are driven by basic meteorological parameters, the meteorological parameters must first be corrected from their observation height to the model reference height (hereinafter referred to as the reference height). Subsequently, the corrected meteorological data are input into the model to compute $T_{m}$ at the reference height. Finally, $T_{m}$ is further corrected from the reference height to the station height. Based on this, this paper proposes a dual vertical correction method. The specific procedure is as follows:

① Correct the temperature to the model reference height. Based on the linear relationship between temperature and height, the site temperature $T^{Z_{site}}$ is vertically corrected to obtain the temperature at the reference height $T^{Z_{ref}}$.

② Calculate the water vapor pressure at the reference height. First, based on the site temperature and relative humidity, use Equations (5) and (6) to calculate the water vapor pressure at the height of the site; then, obtain the surface dew-point temperature $T_{dew}^{z_{site}}$ via the inverse function of Equation (7). Subsequently, according to the linear relationship between dew-point temperature and height, compute the dew-point temperature $T_{dew}^{z_{ref}}$ at the reference height:

(16) $$T_{dew}^{z_{ref}}=T_{dew}^{z_{site}}-\Gamma_{dew}*(z_{ref}-z_{site}),$$

where $\Gamma_{dew}$ is the dew-point temperature lapse rate, with a value of 1.8 K/km [40]. Finally, the water vapor pressure at the reference height $e^{z_{ref}}$ is calculated using Equation (7).

③ Calculate the reference height $T_{m}$. Input the calculated $T^{z_{ref}}$ and $e^{z_{ref}}$ into the $T_{m}$ model to obtain the $T_{m}$ value at the reference height $T_{m}^{Z_{ref}}$.

**Figure 4:**
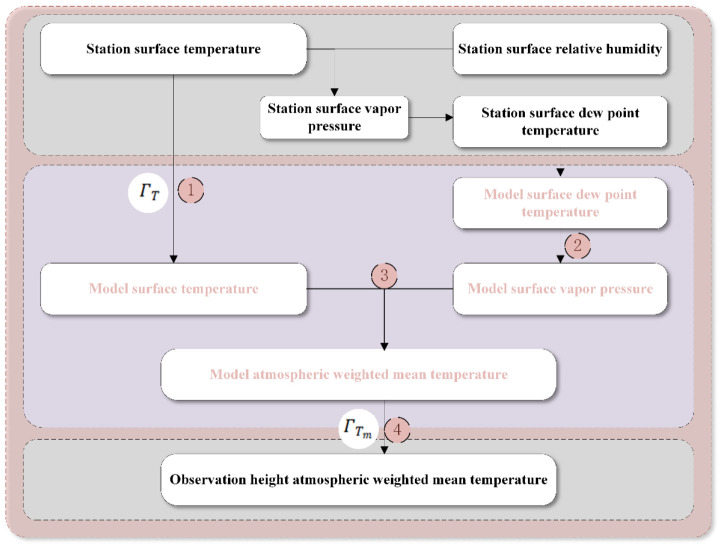
Flowchart of dual vertical correction.

④ Calculate the $T_{m}$ at the GNSS site. Based on the linear relationship between $T_{m}$ and height, and using the $T_{m}$ lapse rate, the reference height $T_{m}^{Z_{ref}}$ is vertically corrected to obtain the site $T_{m}^{Z_{site}}$. The complete workflow of this dual vertical correction method is illustrated in Figure 4.

According to the dual vertical correction method, the vertical correction of temperature in step ④ and the vertical correction of $T_{m}$ in step ④ require the establishment of a temperature vertical lapse rate model and a $T_{m}$ vertical lapse rate model, respectively. To this end, we first extracted the temperature profiles and $T_{m}$ profiles for each grid point from the ERA5 dataset over China for the period 2019–2023. Both temperature and height, as well as $T_{m}$ and height, exhibit a linear relationship:

(17) $$T=m+\Gamma_{T}H,$$

(18) $$T_{m}=n+\Gamma_{T_{m}}H,$$

where *m* and *n* are the fitting intercepts, and $\Gamma_{T}$ and $\Gamma_{T_{m}}$ are the vertical lapse rates of temperature and $T_{m}$, respectively. The least squares method was used to fit Equations (17) and (18) individually, obtaining time-series datasets of $\Gamma_{T}$ and $\Gamma_{T_{m}}$ for each grid point from 2019 to 2023. Finally, following the approach of Böhm et al. [20], these lapse rates were stored in a periodic function form on a $0.25^{\circ }\times 0.25^{\circ }$ grid over China:

(19) $$\Gamma (doy)=A0+A1\cos(\frac{2\pi *doy}{365.25})+B1*\sin(\frac{2\pi *doy}{365.25})+A2\cos(\frac{4\pi *doy}{365.25})+B2*\sin(\frac{4\pi *doy}{365.25}),$$

where Γ denotes either the $\Gamma_{T}$ or the $\Gamma_{T_{m}}$, doy denotes the day of year, $A_{0}$ is the annual mean, $A_{1}$ and $B_{1}$ are the annual amplitudes, and $A_{2}$ and $B_{2}$ are the semi-annual amplitudes. The dual vertical correction method can be applied to both the CGTV-Tm and CGT-Tm models, though for the CGT-Tm model, water vapor pressure correction is unnecessary.

## 4. Results and Discussion

### 4.1. Assessment of the CGTV-Tm Model Driven by Surface-Measured Meteorological Parameters

#### 4.1.1. Comparison to the ERA5 Data

**Figure 5:**
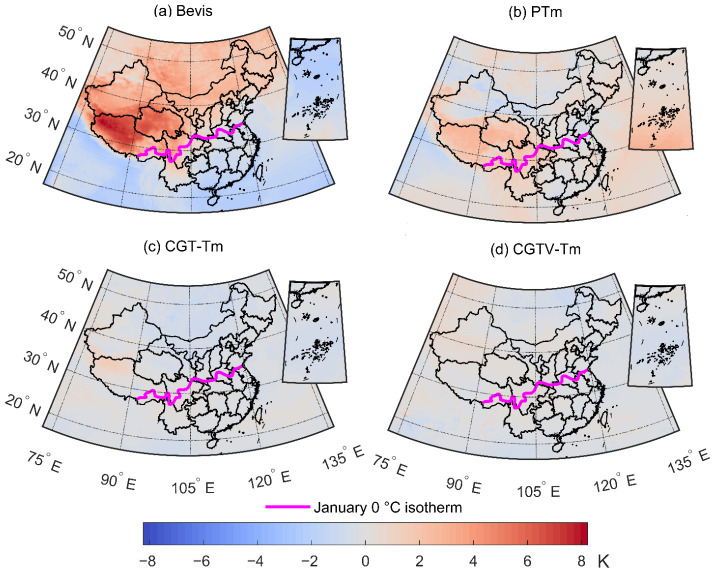
Distribution of bias between $T_{m}$ (atmospheric weighted mean temperature) derived from the four models and ERA5 $T_{m}$.

**Figure 6:**
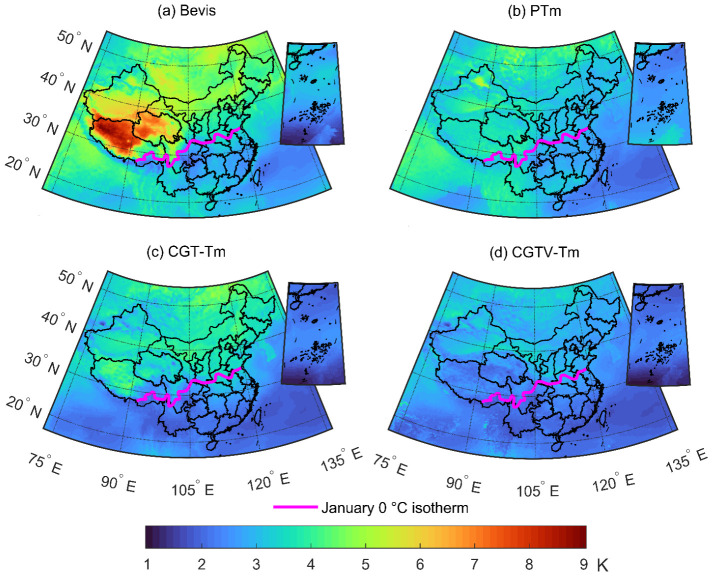
Distribution of RMSE between $T_{m}$ derived from the four models and ERA5 $T_{m}$.

To validate the performance of the CGTV-Tm and CGT-Tm models over China, this study uses the $T_{s}$ and $e_{s}$ data from the 2024 ERA5 reanalysis (which were not involved in modeling) as input sources, and takes the $T_{m}$ values extracted from ERA5 surface and pressure-level data as reference values for independent validation. Comparisons are conducted with the Bevis and PTm models. The statistical results of bias and RMSE for each model are listed in Table 1, while Figure 5 and Figure 6 illustrate the spatial distributions of bias and RMSE, respectively.

The results in Table 1 show that, in terms of bias, the Bevis, PTm, CGT-Tm, and CGTV-Tm models all exhibit positive biases. The Bevis model (mean bias: 0.73 K) and PTm model (mean bias: 0.88 K) show significant systematic biases. The CGT-Tm model has a mean bias of 0.04 K, which is closest to zero, with a bias range of −0.84 to 0.76 K, indicating almost no systematic error. The CGTV-Tm model has a mean bias of 0.08 K and a bias range of −1.48 to 2.14 K, demonstrating good unbiasedness. In terms of RMSE, the Bevis model has the lowest accuracy (mean RMSE: 3.61 K), followed by the PTm model (3.19 K), and the CGT-Tm model further improves accuracy (2.71 K). The CGTV-Tm model performs best, with a mean RMSE of 2.38 K. Compared to the other three models, its accuracy improves by 34.1%, 25.4%, and 12.2%, respectively.

Regarding the spatial distribution of bias and RMSE across models, the Bevis model exhibits the highest overall bias, with significant spatial heterogeneity. Using the January 0 °C isotherm as a boundary, the model shows predominantly positive bias north of the isotherm and negative bias south of it, with a high-bias center located in the Tibetan Plateau (Figure 5a). The spatial distribution of RMSE is generally consistent with that of bias, also demarcated by the January 0 °C isotherm; the RMSE is significantly higher north of the isotherm than south of it, with the Tibetan Plateau being an obvious high RMSE cluster (Figure 6a). This spatial pattern is likely closely related to regional differences in surface elevation and the spatial heterogeneity of atmospheric water vapor content. After incorporating water vapor pressure, the PTm model exhibits improved bias (Figure 5b) and a reduction in overall RMSE. Although the PTm model alleviates the overestimation issues of the Bevis model over the Qinghai–Tibet Plateau, due to its insufficient spatial resolution, it still shows overestimation over the Qinghai–Tibet Plateau, southwestern China, and oceanic regions, while underestimation occurs in northwestern China, resulting in still-pronounced spatial heterogeneity of its RMSE (Figure 6b). Benefiting from its higher spatial resolution, the CGT-Tm model fully refines the influence of topographic variations, further narrowing the bias range (Figure 5c) and effectively reducing the RMSE in complex terrain areas, especially over the Qinghai–Tibet Plateau. However, because this model does not account for water vapor factors, its ability to characterize the vertical atmospheric structure in arid regions is insufficient, resulting in still-significant error differentiation across the January 0 °C isotherm (Figure 6c). The spatial distribution pattern of bias in the CGTV-Tm model is essentially consistent with that of the CGT-Tm model (Figure 5d). Building upon the CGT-Tm model and further incorporating water vapor pressure, the CGTV-Tm model achieves the best RMSE accuracy among the four models, with RMSE values stably concentrated in the range of 1–3 K over most regions and a more uniform spatial distribution of errors (Figure 6d). Specifically, in complex terrain areas such as the Qinghai–Tibet Plateau and the Hengduan Mountains, the RMSE spatial gradient of the CGTV-Tm model is the gentlest, with no significant high-error clustering. The error transitions between coastal and inland areas, as well as between plains and mountains, are continuous and smooth, without obvious boundary effects. At the same time, the model effectively reduces the RMSE difference across the January 0 °C isotherm. The reason is that, while relying on a high spatial resolution to accurately represent elevation differences, the CGTV-Tm model incorporates water vapor pressure, enabling it to better depict the complex atmospheric structure north of the January 0 °C isotherm where water vapor content is relatively low, thereby significantly weakening the error differentiation across the isotherm. Overall, the CGTV-Tm model achieves the smallest RMSE over China, suffers the least interference from spatial heterogeneity factors such as topography, water vapor, and latitude, and demonstrates significantly better accuracy and stability than the other models.

#### 4.1.2. Comparison to the Radiosonde Data

**Figure 7:**
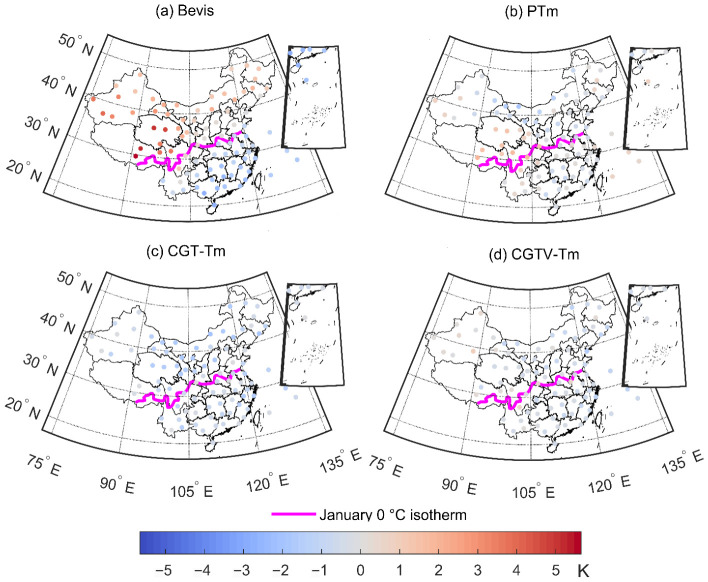
Distribution of bias between $T_{m}$ derived from the four models and the RS $T_{m}$.

**Figure 8:**
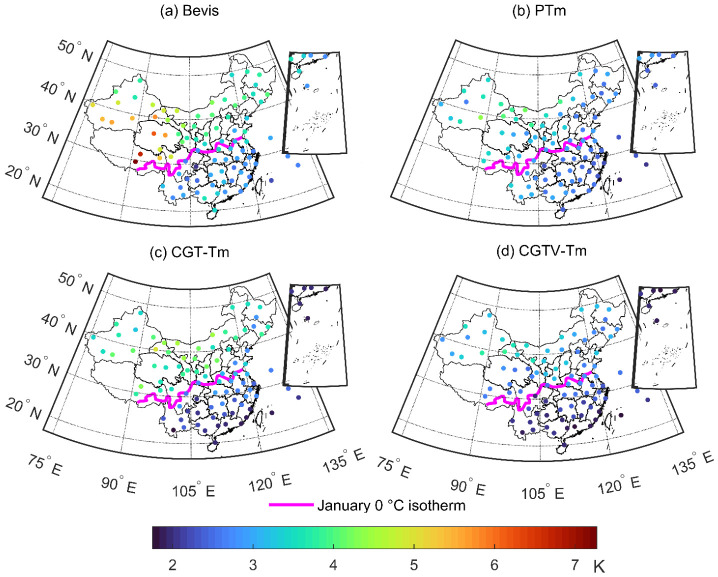
Distribution of RMSE between $T_{m}$ derived from the four models and the RS $T_{m}$.

To further validate the performance of the CGTV-Tm model, this study used $T_{m}$ data from 89 radiosonde stations across China in 2024 as reference values to verify the accuracy of $T_{m}$ calculations by the CGTV-Tm and CGT-Tm models at these stations, and compared them with the Bevis and PTm models. The observed surface data required for $T_{m}$ calculations by these models were all obtained from radiosonde stations. The RMSE and bias values for each model are listed in Table 2, and their spatial distributions are shown in Figure 7 and Figure 8, respectively.

As shown in Table 2, the average bias of the CGTV-Tm model is −0.36 K; among the models analyzed, its absolute value is greater than that of only the PTm model. This may be attributed to the PTm model being directly constructed from radiosonde data, which shares a homologous relationship with the reference radiosonde $T_{m}$, resulting in relatively smaller systematic errors overall. However, the CGTV-Tm model exhibits the smallest bias range, spanning from −1.46 K to 1.02 K, indicating better stability compared to the other models. Additionally, the mean RMSE of the CGTV-Tm model is 2.64 K, which is 0.44 K, 1.03 K, and 0.30 K lower than those of the CGT-Tm, Bevis, and PTm models, respectively, corresponding to relative improvements of 14.3%, 28.1%, and 10.2%. Meanwhile, the RMSE range of the CGTV-Tm model is also the smallest (1.75 K to 3.87 K), further confirming that this model achieves the best accuracy and robustness in the Chinese region.

Figure 7 illustrates that the spatial distribution of the bias for each model is generally consistent with its performance in the comparison with ERA5 data, so it will not be elaborated upon here. It should be noted that although the PTm model has the smallest absolute mean bias (−0.004 K) among all the models, its spatial distribution is not uniform: it exhibits a relatively pronounced negative bias in northwestern China, while showing a significant positive bias over the Qinghai–Tibet Plateau. In contrast, the bias distribution of the CGTV-Tm model is more uniform, demonstrating better stability. In terms of RMSE (Figure 8), the spatial distribution of each model is also consistent with the comparison results of the ERA5 data. Both the Bevis and CGT-Tm models exhibit a spatial distribution pattern of RMSE bounded by the January 0 °C isotherm. Relatively large RMSE values are mostly distributed to the north of the isotherm, while low values are concentrated to the south. After incorporating water vapor pressure, the PTm and CGTV-Tm models break this spatial constraint, with their RMSE values being significantly lower than those of the Bevis and CGT-Tm models, respectively. This further demonstrates that the introduction of water vapor in modeling can effectively reflect the structure and complexity of the atmospheric environment, thereby improving model accuracy. Overall, the comparison with radiosonde data reconfirms that the CGTV-Tm model outperforms the CGT-Tm, Bevis, and PTm models in the Chinese region.

**Figure 9:**
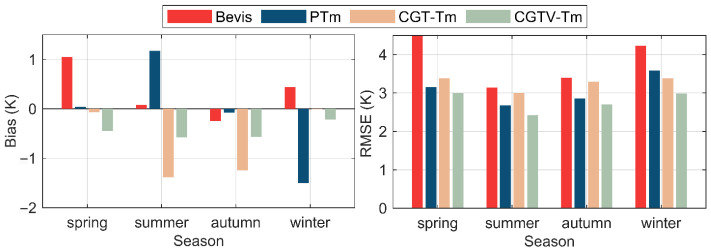
Variation of seasonal RMSE between $T_{m}$ derived from the four models and the RS $T_{m}$.

Given the significant seasonal differences in $T_{m}$, this study further evaluates the applicability of the CGTV -Tm model across different seasons. Figure 9 illustrates the seasonal mean bias and RMSE of the CGTV-Tm, CGT-Tm, PTm, and Bevis models, averaged across 89 radiosonde stations. As shown, the CGTV-Tm model delivers the most stable seasonal bias among the four models, with bias values ranging from −0.6 K to −0.2 K. It also outperforms the other three models with lower RMSE values throughout all seasons, indicating that incorporating water vapor pressure effectively mitigates the impact of seasonal $T_{m}$ variability on estimation accuracy. In contrast, the PTm model suffers from the largest seasonal bias fluctuation, with a summer bias of 1.2 K and a winter bias of −1.5 K. It achieves higher accuracy than the CGT-Tm model in the spring, summer, and autumn, yet yields poorer performance in the winter. This discrepancy may be attributed to the drastic spatiotemporal variations in temperature and water vapor during winter, which induce strong heterogeneity in $T_{m}$ that cannot be accurately captured by the low-spatial-resolution PTm model. The CGT-Tm model maintains a relatively stable seasonal RMSE with a fluctuation of only 0.3 K, yet presents systematic negative biases in the summer (−1.4 K) and autumn (−1.2 K). This finding reveals the limitations of linear models relying only on surface temperature in seasons with high water vapor content. The Bevis model produces relatively large estimation errors in the spring and winter (RMSE > 4.0 K), demonstrating the weakest seasonal adaptability among all the evaluated models. Collectively, this comparison verifies that high-resolution $T_{m}$ models coupling surface temperature and water vapor pressure can effectively improve the seasonal stability of $T_{m}$ estimations.

### 4.2. Assessment of the CGTV-Tm Model Driven by Daily Weather Forecast Data

**Figure 10:**
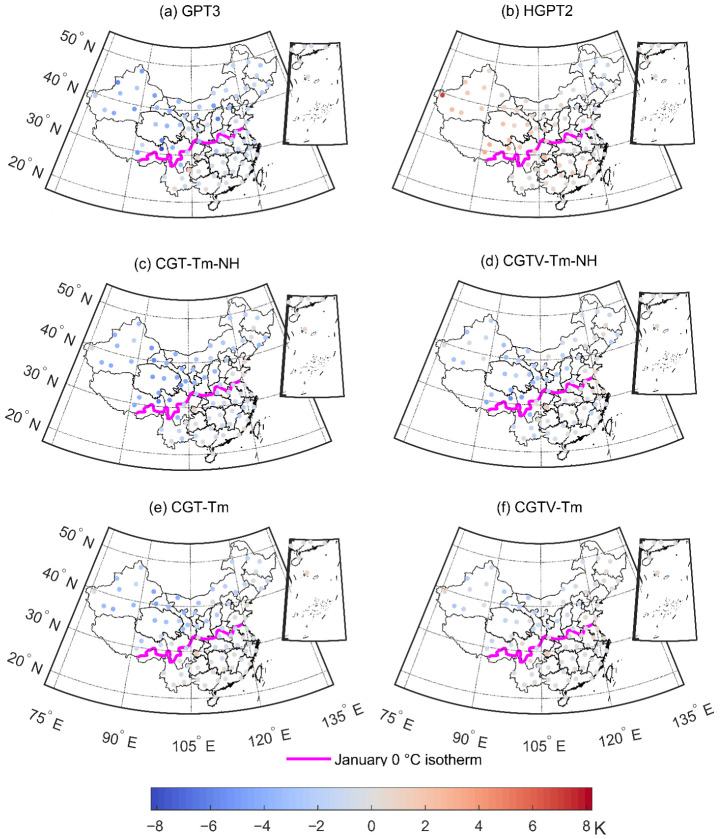
Distribution of bias between the forecasted $T_{m}$ derived from the six models and the RS $T_{m}$.

The core objective of the CGTV-Tm model is to achieve high-accuracy $T_{m}$ estimations by utilizing easily accessible daily weather forecast data, thereby eliminating dependence on in situ meteorological instruments. To this end, this study selects daily weather forecast data from 83 prefecture-level cities provided by the China Meteorological Administration for 2024 as the data source for the CGTV-Tm and CGT-Tm models. Using the $T_{m}$ values computed from 83 radiosonde stations located in these cities during the same period as a reference, we evaluate the accuracy of the new models in real-time forecasting scenarios and compare them with the GPT3 and HGPT2 models. Additionally, to verify the effectiveness of the height correction algorithm, the accuracy of the CGTV-Tm-NH and CGT-Tm-NH models (without height correction) is also assessed. It should be noted that, since the daily weather forecast data contain only surface meteorological parameters and lack height information, the reference height of the CGTV-Tm model is used as the default height for these forecast data. The RMSE and bias values for each model are listed in Table 3, and their spatial distributions are shown in Figure 9 and Figure 10, respectively.

As shown in Table 3, the mean bias of the HGPT2 model is 0.56 K, indicating that its estimated $T_{m}$ values are systematically higher than the reference values, whereas the $T_{m}$ values derived from the other models are all lower than the reference values. The absolute mean biases of the CGT-Tm-NH and GPT3 models are relatively large, reaching 1.97 K and 1.94 K, respectively. After height correction, the absolute bias of the CGT-Tm-NH model decreases to 1.2 K (corresponding to the CGT-Tm model); the mean absolute bias of the CGTV-Tm model decreases from 1.11 K before correction (CGTV-Tm-NH) to 0.34 K, becoming the smallest mean absolute bias among all the models. This result indicates that height correction can effectively reduce the systematic error of the models. The CGTV-Tm model has the smallest bias range, ranging from −2.64 K to 1.32 K. In terms of RMSE, after height correction, the mean RMSE of the CGTV-Tm model decreases from 3.23 K (CGTV-Tm-NH) to 2.90 K; the CGT-Tm model decreases from 3.65 K (CGT-Tm-NH) to 3.22 K, reconfirming the necessity of height correction. The CGTV-Tm model exhibits the smallest mean RMSE and the smallest RMSE range among all the models. Its accuracy is improved by 29.6%, 21.2%, 20.6%, 10.2%, and 9.9% compared to the GPT3, HGPT2, CGT-Tm-NH, CGTV-Tm-NH, and CGT-Tm models, respectively.

Figure 10 shows that GPT3, CGT-Tm-NH, CGTV-Tm-NH, and CGT-Tm mainly exhibit negative bias north of the January 0 °C isotherm and positive bias south of it. In contrast, the HGPT2 model shows a distinctly different pattern, with its bias distribution primarily bounded by 105° E longitude: it shows positive bias west of this meridian and a slight negative bias trend east of it. After incorporating height correction, the CGTV-Tm model drives bias values at stations north of the January 0 °C isotherm, especially over the Qinghai–Tibet Plateau, to gradually approach 0 K, and the overall bias distribution becomes more uniform, demonstrating excellent stability.

**Figure 11:**
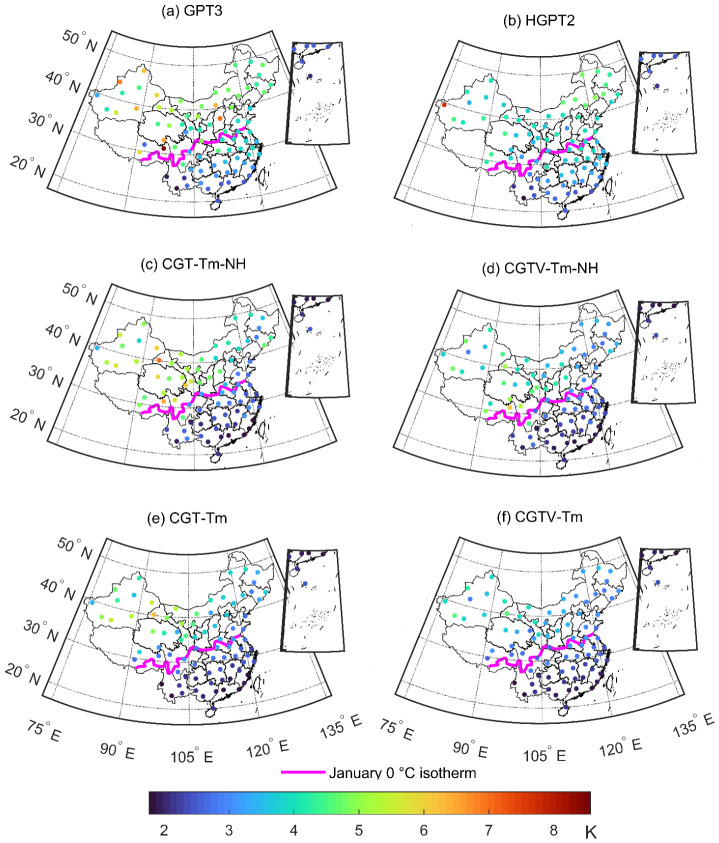
Distribution of RMSE between the forecasted $T_{m}$ derived from the six models and the RS $T_{m}$.

Figure 11 shows that that the RMSE values of all the models exhibit a gradually increasing trend from low to high latitudes. The RMSE values of the GPT3, CGT-Tm-NH, and CGTV-Tm-NH models are clearly demarcated by the January 0 °C isotherm, with larger values mainly appearing north of this isotherm and smaller values south of it. In contrast, the HGPT2 model shows a more gradual change around the January 0 °C isotherm. After height correction, the CGT-Tm and CGTV-Tm models show a further northward shift of the regions with larger RMSE values, with the most significant improvement observed over the Qinghai–Tibet Plateau. Furthermore, after incorporating the water vapor pressure, compared to the CGT-Tm model, the CGTV-Tm model exhibits significantly reduced RMSE values at stations in the arid northwest region, with a more uniform overall distribution: its RMSE values south of the January 0 °C isotherm are typically around 2 K, and the RMSE over most of China is concentrated in the range of 1–3.5 K. In summary, by coupling surface temperature and water vapor pressure and incorporating a dual vertical correction, the CGTV-Tm model not only enables the use of daily weather forecast data as the input, eliminating dependence on in situ meteorological instruments, but also achieves high-accuracy and stable $T_{m}$ predictions.

## 5. Conclusions

$T_{m}$ is a key parameter affecting the accuracy of GNSS PWV retrieval. Existing $T_{m}$ models based on surface-measured meteorological parameters achieve high accuracy but heavily rely on in situ meteorological instruments, leading to high construction and maintenance costs. In contrast, empirical $T_{m}$ models do not depend on measured parameters but struggle to capture short-term variations in meteorological parameters, resulting in relatively low accuracy. This study takes daily weather forecast data—which are low-cost, easily accessible, and capable of reflecting short-term variations in meteorological parameters—as a breakthrough. Focusing on China, we used ERA5 reanalysis data with a spatial resolution of $0.25^{\circ }\times 0.25^{\circ }$ to construct a gridded $T_{m}$ model (namely CGTV-Tm) that couples surface temperature and water vapor pressure, and proposed a corresponding dual vertical correction method. Users only need to input daily weather forecast data to obtain high-accuracy $T_{m}$ estimates in real time. Finally, the CGTV-Tm model was validated using $T_{m}$ values derived from ERA5 reanalysis data and radiosonde data.

(1) Validation against ERA5 and radiosonde data shows that the accuracy spatial patterns of Bevis and CGT-Tm (only using surface temperature) are distinctly bounded by the January 0 °C isotherm. With water vapor pressure introduced, PTm and CGTV-Tm show spatially more stable accuracy nationwide. Benefiting from a higher spatial resolution, CGTV-Tm outperforms PTm across China. Adding water vapor pressure effectively improves model performance in the arid, semi-arid, and complex terrain areas of western China, addressing the limitations of conventional models in high-altitude and dry regions.
(2) In forecast-driven validation, the dual height correction method significantly improves CGTV-Tm’s and CGT-Tm’s accuracy over their uncorrected versions. CGTV-Tm achieves an RMSE of 2.90 K, an improvement of 29.6% and 21.2% over GPT3 (4.12 K) and HGPT2 (3.68 K), respectively. Further refinement of forecast data resolution (e.g., from prefecture-level to county-level or even finer) is expected to enhance CGTV-Tm’s accuracy.
(3) With only publicly available daily weather forecast data as the input, the CGTV-Tm model delivers real-time high-accuracy $T_{m}$ predictions, offering a superior alternative to classic empirical models for GNSS stations without in situ meteorological observations and demonstrating extensive application potential.

Since $T_{m}$ is the critical conversion factor between ZWD and PWV, any improvement in its estimation accuracy directly reduces the systematic and random errors in the retrieved PWV. However, this study has only verified the reliability of CGTV-Tm in $T_{m}$ calculations; it has not yet been applied to actual GNSS PWV retrieval tasks. Therefore, future work will focus on the application of the CGTV-Tm model to operational GNSS PWV retrieval across the Chinese mainland. Specifically, we will utilize the tropospheric delay products provided by the Crustal Movement Observation Network of China (CMONOC), in conjunction with the CGTV-Tm model, to retrieve PWV at GNSS stations over China. The retrieved PWV time series will be rigorously compared and validated against PWV observations from collocated radiosonde stations, with particular emphasis on evaluating their performance under extreme weather conditions.

## Figures and Tables

**Table 1:** Statistical comparison of RMSE (root mean square errors) and bias between $T_{m}$ (atmospheric weighted mean temperature) values derived from the Bevis, PTm, CGT-Tm, and CGTV-Tm models and those derived from ERA5 data. All four models were driven by surface meteorological parameters from ERA5.

Models	RMSE (K)			Bias (K)		
	Mean	Min	Max	Mean	Min	Max
Bevis	3.61	1.11	8.46	0.73	−3.12	7.39
PTm	3.19	1.73	5.69	0.88	−2.40	3.71
CGT-Tm	2.71	0.93	4.72	0.04	−0.84	0.76
CGTV-Tm	2.38	0.83	4.30	0.08	−1.48	2.14

**Table 2:** Statistical comparison of RMSE and bias between $T_{m}$ values derived from the Bevis, PTm, CGT-Tm, and CGTV-Tm models and those derived from radiosonde data. All four models were driven by surface meteorological parameters from radiosonde stations.

Models	RMSE (K)			Bias (K)		
	Mean	Min	Max	Mean	Min	Max
Bevis	3.67	2.07	7.27	0.39	−2.66	5.66
PTm	2.94	1.98	4.38	−0.004	−1.91	1.86
CGT-Tm	3.08	1.84	4.95	−0.64	−1.95	1.20
CGTV-Tm	2.64	1.75	3.87	−0.36	−1.46	1.02

**Table 3:** Statistical comparison of RMSE and bias between $T_{m}$ values derived from the GPT3, HGPT2, CGT-Tm-NH, CGTV-Tm-NH, CGT-Tm, and CGTV-Tm models and those derived from radiosonde data. All six models were driven by surface meteorological parameters from daily weather forecast data.

Models	RMSE (K)			Bias (K)		
	Mean	Min	Max	Mean	Min	Max
GPT3	4.12	1.78	8.56	−1.94	−8.21	1.84
HGPT2	3.68	1.85	7.68	0.56	−1.33	7.14
CGT-Tm-NH	3.65	1.77	6.99	−1.97	−5.95	1.40
CGTV-Tm-NH	3.23	1.79	6.12	−1.11	−5.45	1.67
CGT-Tm	3.22	1.79	6.25	−1.20	−4.74	1.05
CGTV-Tm	2.90	1.81	4.55	−0.34	−2.64	1.32

## Data Availability

The raw data supporting the conclusions of this article will be made available by the authors on request.
